# A Simple Approach to Characterize Sorption and Release Kinetics in Polymeric Materials with Planar, Cylindrical or Spherical Geometries

**DOI:** 10.3390/polym17243298

**Published:** 2025-12-12

**Authors:** Sara Exojo-Trujillo, Laura Higueras-Contreras, Carol López-de-Dicastillo, Pilar Hernández-Muñoz, Rafael Gavara

**Affiliations:** 1 Packaging Group, Instituto de Agroquímica y Tecnología de Alimentos, CSIC, C/Agustin Escardino 7, 46980 Paterna, Spain; saetru@iata.csic.es (S.E.-T.); lauhicon@iata.csic.es (L.H.-C.); clopezdedicastillo@iata.csic.es (C.L.-d.-D.); phernan@iata.csic.es (P.H.-M.); 2 PhD in Food Science, Technology and Management, Universitat Politècnica de València, Camino de Vera s/n, 46022 Valencia, Spain

**Keywords:** Fick’s laws, sorption, release, finite-difference method, film, fibers, spheres

## Abstract

This study presents a theoretical framework for modeling sorption and release kinetics of substances in polymeric materials with planar, cylindrical, and spherical geometries. Fick’s second law was expressed in dimensionless variables and solved numerically using a finite-difference approach to generate universal profiles for mass transfer. These profiles were fitted with double-exponential equations, yielding explicit expressions that allow for straightforward estimation of diffusion coefficients from experimental data. The method was validated using literature data for films, fibers, and microspheres, showing excellent agreement with reported values. Unlike classical analytical solutions, which are limited to planar systems under ideal conditions, the proposed approach is applicable to diverse geometries commonly employed in packaging, biomedical devices, controlled-release formulations, and environmental technologies.

## 1. Introduction

Polymers are widely used across all industrial sectors due to their versatile mechanical, optical, thermal, and barrier properties. Among these, mass transfer characteristics—namely permeability, sorption, and migration—play a crucial role in determining the performance and functionality of polymeric materials in many applications [1]. These properties govern the interaction between the polymer matrix and external substances, including gases, vapors, and low-molecular-weight compounds, which are highly relevant in applications such as packaging, pharmaceutical and medical devices, separation membranes, electronic encapsulation, controlled release of fertilizers or pesticides, and remediation of contaminated soils and water.

While traditionally considered undesirable in applications such as in food packaging due to potential contamination risks, sorption and migration phenomena can be harnessed positively [2]. For instance, they enable the retention of substances such as volatile organic compounds, ethylene, pesticides and other contaminants, as well as the controlled release of active agents such as antimicrobials, antioxidants, fertilizers or aroma compounds. These functionalities are central to the development of active and intelligent packaging systems, and novel products for medicine, agriculture or environment [3,4,5,6].

The theoretical description of these processes is typically based on Fick’s laws of diffusion, which relate the flux of a substance to its concentration gradient, and Henry’s law, which governs the equilibrium partitioning between phases. However, solving Fick’s second law analytically in real systems is often challenging due to complex geometries, boundary conditions, and time-dependent behaviors [7]. This difficulty hampers the direct estimation of the diffusion coefficient, a key parameter that characterizes the rate of mass transfer within the polymer matrix.

To overcome these limitations, researchers have developed simplified models using idealized geometries such as planar sheets, cylindrical rods, and spherical particles. These geometries allow for tractable analytical or semi-analytical solutions to Fick’s equations, facilitating the interpretation of experimental sorption/desorption kinetics and the estimation of diffusion coefficients under controlled conditions. Nevertheless, explicit analytical solutions to Fick’s law are available only for planar geometries and only under ideal boundary conditions [8].

In numerous scientific and industrial applications, the use of small particles and fibers has become increasingly prevalent due to their high surface-area-to-volume ratios and tunable transport properties [9]. These structures are often engineered from polymers, composites, or hybrid materials, and their geometries—whether spherical or cylindrical—lend themselves to simplified mathematical modeling of mass transfer processes. Spherical particles, for instance, are commonly employed in adsorbent systems [10], controlled release formulations [11], and sensor technologies, where radial diffusion of molecules governs sorption and desorption kinetics.

The fabrication of microparticles with near-spherical geometry is a common strategy in fields such as drug delivery, catalysis, and environmental remediation. Techniques such as spray drying, emulsion polymerization, and solvent evaporation are widely employed to produce polymeric microspheres with controlled size and morphology [12]. These particles serve as carriers for active substances, adsorbents for pollutants, or scaffolds for biological applications. Their spherical shape simplifies the mathematical treatment of mass transfer, enabling the use of radial diffusion models to describe sorption and release kinetics.

Similarly, fibers and filaments, which can be approximated as cylindrical geometries, are widely used in textile engineering, filtration, and drug delivery systems. Their elongated shape facilitates directional diffusion and can be exploited to create anisotropic transport profiles. In both cases, the adoption of idealized geometries enables the application of Fick’s laws under well-defined boundary conditions, allowing researchers to extract meaningful diffusion coefficients and better understand the dynamic behavior of substances within these materials.

In parallel, the development of microfibers and nanofibers, which can be approximated as cylindrical structures, has gained prominence in applications ranging from filtration and tissue engineering to smart textiles and sensors. Electrospinning is one of the most versatile and widely used methods for producing polymeric fibers with diameters ranging from tens of nanometers to several micrometers [13]. Other techniques include melt spinning, wet spinning, and dry spinning, depending on the polymer type and desired fiber properties [14]. These fibers offer directional diffusion pathways and high aspect ratios, making them ideal for controlled transport of molecules along their length.

Unlike widely used empirical models such as the Korsmeyer–Peppas power law, which classify release mechanisms based on an arbitrary diffusional exponent (*n*) without providing physically meaningful parameters, the approach proposed in this work delivers explicit analytical expressions for estimating the diffusion coefficient (*D_i_*). This parameter is fundamental for characterizing mass transfer in polymeric systems and enables a mechanistic interpretation of sorption and release processes across planar, cylindrical, and spherical geometries. By overcoming the limitations of semiempirical models—whose applicability is restricted and whose parameters lack direct physical significance—our method offers a universal and robust framework that facilitates accurate prediction, comparison, and design of functional polymer-based systems. This advantage is particularly relevant in applications such as active food packaging, where controlled release of antioxidants or antimicrobials is critical [6], and in drug delivery systems, where precise diffusion control determines therapeutic efficacy [7]. Unlike empirical approaches such as Weibull or Peppas–Sahlin models, which rely on curve fitting and provide no direct link to material properties, our methodology enables the estimation of *D_i_* from experimental data under realistic conditions, supporting rational design and optimization of packaging, biomedical devices, and environmental technologies [8,9].

In this work, explicit solutions for the sorption and release of substances in polymeric materials with planar, cylindrical or spherical geometries were developed and a simple procedure for the estimation of the diffusion coefficient values of a sorbate within the polymer matrix is provided, enabling the modeling of these mass transfer processes. The novelty of this study lies in providing universal, explicit expressions derived from Fick’s second law for sorption processes. This approach enables a straightforward and accurate estimation of diffusion coefficients from experimental data, overcoming the limitations of classical analytical solutions restricted to planar system and the lack of meaning of the empirical model parameters.

## 2. Theoretical Background

Mass transport though solid media such as polymeric materials is of critical importance in a wide range of technological applications. Properties such as permeability, sorption, and migration govern the movement of substances into, through, and out of polymer matrices, influencing both the performance and safety of the final product. The theoretical description of these processes is commonly based on Fick’s laws of diffusion and the partition equilibrium (Henry’s law if the fluid phase is gaseous), which together provide a framework for understanding the kinetics and equilibrium behavior of molecular transport in polymers [2]. Henry’s constant provides an estimate of the equilibrium concentrations of a substance between the two phases, the solid polymer and the surrounding medium (liquid or gas).

Equilibrium concentrations are easily obtained by exposing the polymer to a fluid containing the transferred substance in any or both phases, and measuring its concentration in both polymer and fluid. At low concentrations, these ratio between equilibrium concentrations is generally constant and can be defined by the partition coefficient (*K_i_*) or the solubility coefficient (*S_i_*):

(1) $$K_{i}\;=\;\frac{c_{i}^{p}}{c_{i}^{F}}\;;\;S_{i}\;=\;\frac{c_{i}^{p}}{p_{i}^{F}}$$

where $c_{i}^{p}$ and $c_{i}^{F}$ are the substance *i* concentrations in the polymer and in the fluid, respectively, and $p_{i}^{F}$ the partial pressure of *i* in the gaseous fluid. At high concentrations deviations from linearity may occur as a result of matrix plasticization. Although in semicrystalline polymers only amorphous regions are available for sorption and retention of penetrants, the polymeric material is usually considered homogeneous and an apparent value for the partition equilibrium is provided. Nevertheless, values of these parameters vary with the crystallinity percentage, the greater the crystallinity the lower the value of *K_i_* or *S_i_*.

To estimate the sorption or release kinetics, Fick’s laws of diffusion are normally considered. Fick’s first law states that a solute moves from a region of high concentration to a region of low concentration across a concentration gradient. In one dimension (*x*), the law can be written as:

(2) $$J_{i}\;=\;-D_{i}\cdot \frac{dc_{i}}{dx}$$

where *J_i_* is the diffusion flux, expressed as the amount of substance (in mass or volume) per unit area and unit time, *D_i_* is the diffusion coefficient or diffusivity (in area per unit time), and *dc_i_*/*dx* is the concentration gradient [2].

Fick’s second law describes how diffusion causes the concentration to change with respect to time. This law can be expressed for diffusion in one dimension as:

(3) $$\frac{dc_{i}}{dt}\;=\;\frac{\partial }{\partial x}\left(D_{i}\cdot \frac{dc_{i}}{dx}\right)$$

Considering that the materials is homogeneous and *D_i_* is independent of position, the second law can be simplified to:

(4) $$\frac{dc_{i}}{dx}\;=\;D_{i}\cdot \frac{\partial^{2}c_{i}}{\partial x^{2}}$$

This assumption represents an idealized condition for several reasons. Most polymeric materials are semicrystalline, consisting of crystalline spherulites dispersed within an amorphous matrix. Consequently, the diffusion coefficient (*D_i_*) is actually an apparent value that may vary in particles made from the same polymer but exhibiting different degrees of crystallinity [15]. Another important factor is the effect of swelling or plasticization. In many experiments, the sample undergoes swelling and plasticization due to the sorption of low-molecular-weight species—often the solvent, migration simulant, or moisture [16]. When the sorption of this plasticizing agent occurs much faster than the release or sorption of the solute, Equation (4) can be considered valid, as the solute diffuses through the plasticized matrix [17]. However, if plasticization (or antiplasticization) results from the sorption (or desorption) of the solute itself, Equation (4) becomes invalid, and the progression of the mass transfer process will differ from that described in this work.

Several solutions to Equations (2) and (4) have been obtained under ideal boundary conditions, allowing the estimation of the diffusion coefficient and, therefore, the characterization of sorption or release processes. For instance, considering an infinite film of constant thickness (l) exposed by both sides to an infinite solution with a constant concentration of the solute ($c_{i,s}^{\infty }$), Equation (4) can be analytically solved and explicitly expressed as [8]:

(5) $$\frac{M_{i,t}}{M_{i,\infty }}\;=\;1-\sum_{n=0}^{\infty }\frac{8}{{\left(2n\;+\;1\right)}^{2}\cdot \pi^{2}}exp\left(-\frac{D_{i}\cdot {\left(2n\;+\;1\right)}^{2}\cdot \pi^{2}\cdot t}{l^{2}}\right)$$

where *M*_*i*,*t*_ and *M*_*i*,*∞*_ are the solute mass gained by the film at time *t* and at equilibrium, respectively. This latter can be estimated if the partition coefficient of the solute between the two phases (polymer and fluid), *K_i_*, is known:

(6) $$M_{i,\infty \;}=\;c_{i,p}^{\infty }\cdot l\cdot A\;={\;K}_{i}\cdot c_{i,F}^{\infty }\cdot l\cdot A$$

Also, an explicit analytical solution has been obtained for a film exposed to a finite solution of initial concentration ($c_{i,F}^{0}$), where the limited solute is partitioned between two phases:

(7) $$\frac{M_{i,t}}{M_{i,\infty }}\;=\;1-\sum_{n=0}^{\infty }\frac{2\alpha (\alpha \;+\;1)}{\alpha (\alpha \;+\;1)\cdot q_{n}^{2}}exp\left(-\frac{D_{i}\cdot q_{n}^{2}\cdot t}{l^{2}}\right)$$

where *α* is dimensionless parameter that considers the ratio between the solution volume and the film sorption capacity and *q_n_* values are the positive roots of the equation:

(8) $$\alpha \;=\;\frac{V_{L}}{K_{i}\cdot A\cdot l};\;tan\;\left(q_{n}\right)\;=-\alpha \cdot q_{n}$$

However, no analytical solutions have obtained for Equation (4) for other geometries, particularly spherical and fibrillar polymeric materials, which are widely used in many fields.

## 3. Results and Discussion

As described in the previous section, analytical solutions to Fick’s laws are only available for a limited set of boundary conditions. For other systems, mathematical solutions can be derived, and one of the most useful approaches is the application of the finite-difference method. To implement this, it is useful to express Fick’s laws in dimensionless variables:

(9) $$X\;=\;\frac{x}{l};\;\;T\;=\;\frac{D_{i}\cdot t}{l^{2}};{\;\;C}_{i}\;=\;\frac{c_{i}}{c_{i}^{0}};\;\;\;\frac{dC_{i}}{dX}\;=\;\frac{\partial^{2}C_{i}}{\partial X^{2}}$$

where $c_{i}^{0}$ is a standard or reference concentration selected as relevant for the process, such as the concentration on the surface of the material in a sorption process or the initial concentration in the material in a release process. It is important to note that the methodology presented does not describe or model the equilibrium sorption process. As widely reported, the greater the chemical affinity between the penetrant and the polymer, the higher its equilibrium uptake within the polymer. In this approach, the concentration of the polymer at equilibrium is treated as an experimental value, represented by $c_{i}^{0}$. The methodology focuses exclusively on the kinetics of sorption or desorption, expressed in terms of the relative concentration *C_i_*. In cases where significant sorption of the penetrant or solvent occurs, plasticization of the polymer matrix may take place, altering the diffusion behavior during the test. Since the proposed model assumes a constant diffusion coefficient (*D_i_*), the value obtained under such conditions should be considered an apparent or average diffusion coefficient rather than an absolute one.

### 3.1. Materials with Planar Geometry

In a planar geometry, molecular diffusion occurs along the film thickness, from the two surfaces towards the center in sorption processes or from the center towards both surfaces in release processes. For sorption, $c_{i}^{0}$ corresponds to the final concentration in the material, and for release processes, $c_{i}^{0}$ would be the initial solute concentration in the material. The position *X* along the film thickness is divided into *n* equal slides of thickness *δX*. Time (in dimensionless units) increases in equal *δT* intervals. Applying Taylor’s series under certain assumptions [8], the concentration of sorbate in the *i* slide after *j* + 1 *δT* time steps can be expressed as:

(10) $$C_{i,j+1}-C_{i,j}=\frac{\delta T}{{\left(\delta X\right)}^{2}}\left(C_{i+1,j}-2C_{i,j}\;+\;C_{i-1,j}\right),\;i\neq 0,\;n$$

For a sorption process, the film initially contains no sorbate, and from the process start, the surface concentrations are identical and remain constant:

(11) $$C_{0,0}\;={\;C}_{n,0}\;=\;0,\;T\;=\;0;\;C_{0,j}\;={\;C}_{n,j}\;=\;1,\;T\;>\;0$$

**Figure 1:**
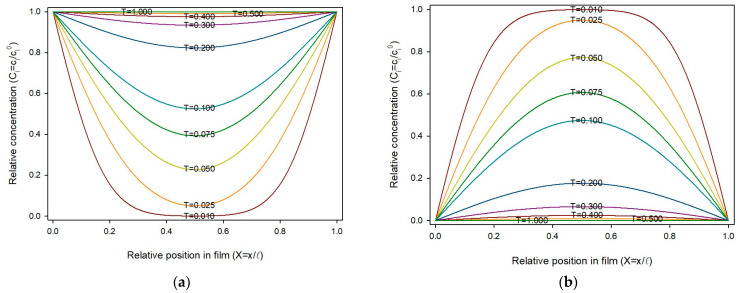
Concentration profile of sorbate *i*, through the film thickness and over time in dimensionless variables: (**a**) Sorption process; (**b**) Release process.

To obtain a suitable description of the process using Equations (10) and (11), the ratio $\delta T/{\left(\delta X\right)}^{2}$ should be ½ or less, otherwise oscillations occur, making the numerical analysis unstable. In this work, δX was taken as 0.02, this is, the thickness was divided into 50 slides, and δT was 0.0001, giving a $\delta T/{\left(\delta X\right)}^{2}$ ratio of 0.25. Figure 1a shows the sorption profiles across the film thickness over time. As can be seen, the sorption progresses symmetrically from the external surface towards the film center. Since *C_i_*, *X* and *T* are dimensionless variables, this profile is universal for homogeneous planar materials under the stated assumptions (constant diffusion coefficient, and fixed boundary conditions). Figure 1b shows similar profiles for a release process.

By summing the sorbate present in each slide at a given exposure time, the relative sorption of solute *i* in the film can be obtained:

(12) $$\frac{m_{i,\;t}}{m_{i,\infty }}=\sum_{i=0}^{n}C_{i,t}\cdot \delta X$$

**Figure 2:**
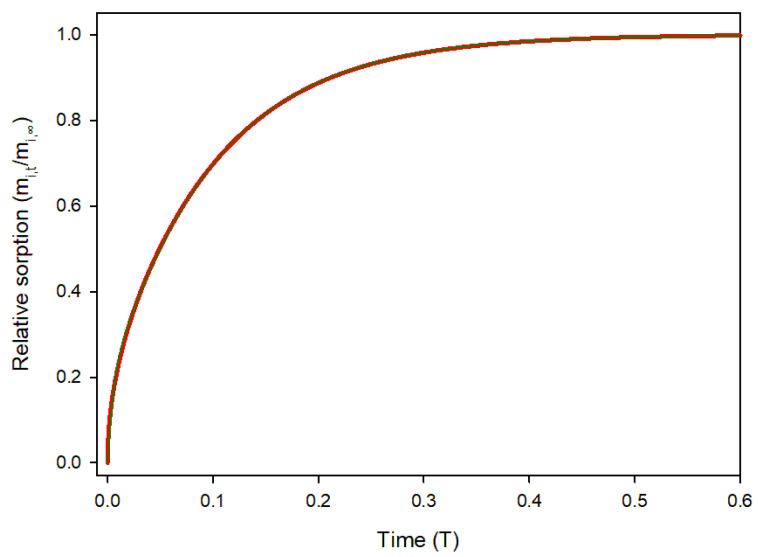
Relative sorption of solute *i* in a film over time in dimensionless variables. Red line: Equation (12); Green line: curve fitting.

The universal sorption profile for a planar material is shown in Figure 2.

Sorption starts rapidly due to a high concentration gradient, and slows as the material saturates. Sorption values were computed for 10,000 time steps (*δT* = 0.0001) and the obtained data were curve fitted using the fitting tool of Sigmaplot v16 with the “Exponential rise to maximum, double, 4-parameter” model [18].

(13) $$\frac{m_{i,\;t}}{m_{i,\infty }}=\alpha \left(1-e^{-\beta \left(\frac{D_{i}t}{l^{2}}\right)}\right)+\gamma \left(1-e^{-\varepsilon \left(\frac{D_{i}t}{l^{2}}\right)}\right)$$

The fit was excellent (R^2^ = 0.99993) with the data being virtually indistinguishable, as shown in Figure 2. The resulting equation was:

(14) $$\frac{m_{i,\;t}}{m_{i,\infty }}\;=\;0.177\left(1-e^{-215.5\left(\frac{D_{i}t}{l^{2}}\right)}\right)\;+\;0.823\left(1-e^{-10.002\left(\frac{D_{i}t}{l^{2}}\right)}\right)$$

This expression explicitly relates the relative gain of solute *i* in the film to time with only one unknown, *D_i_*. Thus, by converting the gain data ($m_{i,\;t}$) to relative values using the estimated equilibrium gain ($m_{i,\infty }$), expressing time in dimensionless form with the film thickness and an initial guess value for *D_i_*, and iterating numerically, the value of *D_i_* can be estimated. In this work, the Excel Solver tool (Microsoft Office 16) was used to obtain an estimation of *D_i_*, by minimizing the summation of squared differences between experimental data and Equation (14).

**Figure 3:**
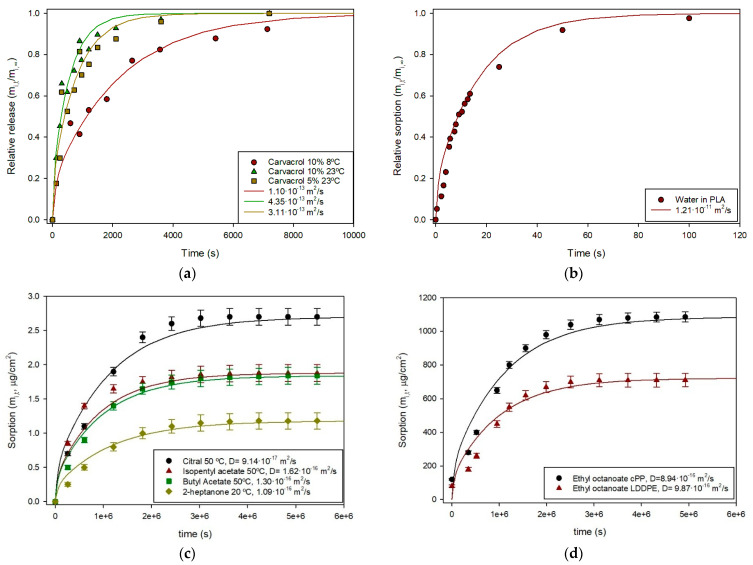
(**a**) Carvacrol release from 50-µm cellulose acetate films at two concentrations and temperatures, experimental data reported [19] and curves obtained from Equation (14); (**b**) Relative water sorption in PLLA film at 23 °C, experimental data reported [20] and curves obtained from Equation (14); (**c**) sorption of four aroma compounds in a PP film laminated to tinplate, experimental data reported [22] and curves obtained from Equation (14); (**d**) sorption of ethyl octanoate in cPP and LLDPE films, experimental data reported [21] and curves obtained from Equation (14).

As examples, Laroque et al. reported the release of carvacrol from cellulose acetate films containing 5% and 10% of this phenolic compound into ethanol at 23 °C, and the 10% films at 8 °C, obtaining *D_i_* values of 1.269·10^−13^, 3.624·10^−13^ and 3.365·10^−13^ m^2^/s, respectively, using Equation (5) [19]. These experimental data were analyzed using Equation (14) following the protocol described, and the fitting curves are shown in Figure 3a, demonstrating a good agreement. Similarly, the water retention in a 46-µm poly(L-lactic acid (PLLA) film was measured and the kinetic characterized by using Equation (5), with a *D_i_* value of 1.1·10^−11^ m^2^/s [20]. The experimental data were collected and the fitting to Equation (14) yielded similar results (Figure 3b). The potential scalping of wine aroma by polymers was also analyzed by measuring the retention of ethyl octanoate in two films, cPP and LLDPE [21], and the results were accurately fitted by Equation (14) as shown in Figure 3c. A similar study was conducted with various aroma compounds in a tinplate laminated with a PET film. In this case, sorption occurs only through one surface, and the film thickness should therefore be considered as double in Equation (14). Figure 3d illustrates the excellent fit obtained for four aroma compounds with diffusion coefficient values in good agreement with the original publication [22].

### 3.2. Materials with Cylindrical Geometry

A fiber can be considered a cylindrical object of infinite length and constant radius (*a*). The second Fick’s law for a material in which substances are sorbed or released in a radial form can be written as:

(15) $$\frac{\partial C_{i}}{\partial T}\;=\;\frac{1}{R}\frac{\partial }{\partial T}\left(R\frac{\partial C_{i}}{\partial R}\right)\;=\;\frac{\partial^{2}C_{i}}{\partial R^{2}}\;+\;\frac{1}{R}\frac{\partial C_{i}}{\partial R}$$

where *R* represents the relative position within the cylinder, defined as the actual distance from the axis (*r*) over the fiber radius *a*. With respect to time, the dimensionless variable *T* is given by a relationship among time, the diffusion coefficient of the sorbate in the polymer and the fiber radius, according to:

(16) $$R\;=\;\frac{r}{a};T\;=\;\frac{D_{i}t}{a^{2}}$$

Unfortunately, there is no explicit solution for the above differential Equation (15). However, numerical solutions can be obtained using a finite-difference approach, dividing the matrix into equal radial intervals (*δR*) in the space 0 ≤ *R* ≤ 1, and the time into regular intervals (*δT*). The concentrations at a point (*iδR*) and at a time (*j* + 1) *δT* can be calculated from the concentration values at that point and the adjacent points, (*i* − 1) *δR* and (*i* + 1) *δR*, in the precedent time (*jδT*) as follows [8]:

(17) $$\frac{c_{i,j+1}-c_{i,j}}{\delta T}\;=\;\frac{1}{2i{\left(\delta R\right)}^{2}}\left(\left(2i\;+\;1\right)c_{i+1,j}-4ic_{i,j}\;+\;\left(2i-1\right)c_{i-1,j}\right),\;i\neq 0$$

With boundary conditions:

(18) $$C_{0,0}\;=\;0,T\;=\;0;\;C_{0,j}\;=\;1,T\;>\;0$$

**Figure 4:**
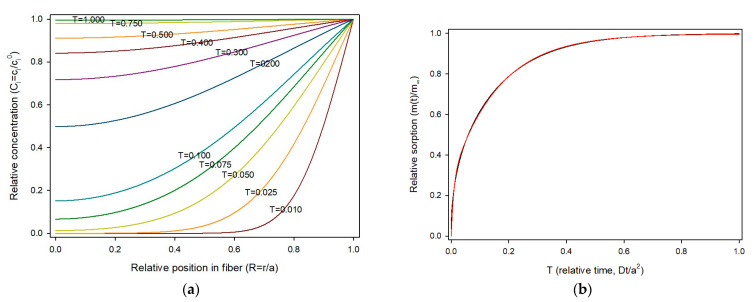
(**a**) Concentration profile of sorbate *i*, across the fiber radius and over time in dimensionless variables; (**b**) Relative sorption in a fiber over time in dimensionless variables. Red line: Equation (17); green line: curve fitting.

Equation (17) was used to compute the theoretical evolution of sorbate concentration in a fiber. For calculations, the fiber radius was divided in 50 parts (*δR* = 1/50 = 0.02). The smaller the finite element for time (*δT*), the better the estimation, but the calculation time increases. To avoid fluctuations, *δT* should be *δT* < 0.5·*δR*^2^ [8]. Thus, the maximum value for *δT* in this work was 0.0002 and *δT* = 0.0001 was selected. Concentrations at each *iδR* from 0 to 1 (axis to surface) for times ranging from 0 to 1 were obtained and the sorption profiles at various relative times are shown in Figure 4a.

As shown, sorption starts rapidly from the fiber surface towards the center due to the high concentration gradient, and slows as the concentration of the solute in the material builds up. The amount of sorbate present in the fiber can be calculated by summing of the quantities of substance present in the circular crown area limited by (*i* + 1)*δR* and *iδR*, as expressed in Equation (19).

(19) $$\frac{m_{i,\;t}}{m_{i,\infty }}=\sum_{i=0}^{n-1}\left(C_{i,t}\cdot \pi \left(R_{i+1}^{2}-R_{i}^{2}\right)\right)$$

Thus, the universal sorption profile for a cylindrical material is presented in Figure 4b. Values of sorption were obtained for 10,000 time steps (*δT* = 0.0001) and the obtained data fitted using the fitting tool of Sigmaplot v16 with the “Exponential rise to maximum, double, 4 parameter” model, as carried out for the planar geometry. The fit was excellent, with a R^2^ = 0.99993, as shown in Figure 3b. The resulting equation was:

(20) $$\frac{m_{i,\;t}}{m_{i,\infty }}\;=\;0.2607\left(1-e^{-130.9\left(\frac{D_{i}t}{l^{2}}\right)}\right)\;+\;0.7241\left(1-e^{-6.589\left(\frac{D_{i}t}{l^{2}}\right)}\right)$$

This expression presents explicitly the relative gain of solute *i* in a fiber as a function of time with only one unknown, *D_i_*. Thus, converting the gain values ($m_{i,\;t}$) in relative values by estimating the equilibrium gain $\left(m_{i,\infty }\right)$, and converting the time in dimensionless time by using the fiber radius and a guess value for *D_i_*, by numerical iteration, the value of *D_i_* can be estimated. Again, the Excel Solver tool of (Microsoft Office 16) was used to obtain an estimation of *D_i_*, by minimizing the summation of squared differences between experimental data and Equation (20).

**Figure 5:**
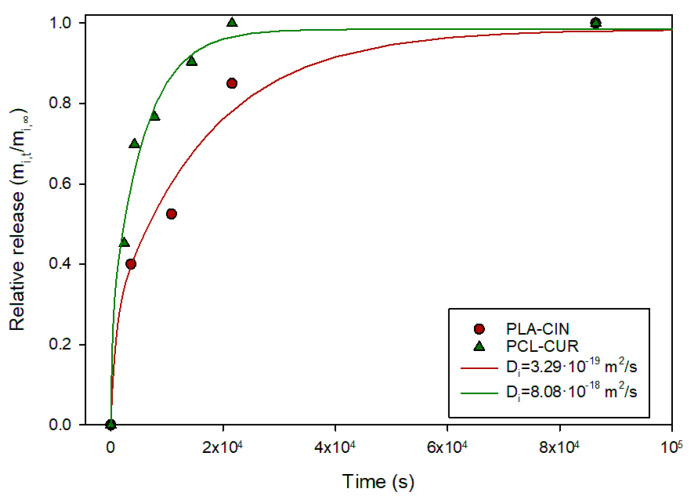
Relative release of cinnamaldehyde and curcumin from 384 nm PLA fibers and 1.12-µm PCL fibers into 10% ethanol.

To test the equation and the protocol exposed, data from two earlier works were analyzed. Mats of polycaprolactone (PCL) fibers containing curcumin were exposed to 10% ethanol and the process kinetics were characterized, with a *D_i_* values reported in the 10^−15^ m^2^/s range [18]. However, in that report, the mats were treated as films (~250 µm thick) rather than fiber mats (fiber diameter: 1.12 µm). Figure 5 shows the collected data from the report, and the fitting to Equation (20), showing that the fitting accurately described the release process, yielding a much lower diffusion coefficient. A similar result was obtained for mats of PLA fibers (diameter: 0.384 µm) containing cinnamaldehyde [18,19]. Again, Equation (20) provided a good fit, considering that the cinnamaldehyde is released from the individual fibers.

Hence, this model could provide a more realistic representation of mass transport in polymeric fibers, such as those in textile materials, compared to treating the fabric as a planar material. Nevertheless, mats of thin fibers prepared by techniques such as electrospinning often cannot be considered as single fibers, since the fibers touch each other and may be interconnected. Although these materials should not be regarded as thick films (as is commonly assumed when analyzing mass transport), applying this model to them may still represent a simplification of the actual scenario.

### 3.3. Materials with Spherical Geometry

Many materials are prepared as microparticles with similar dimensions in all directions. Often, they can be considered as spheres of radius (*a*). The second Fick’s law for a material in which substances are sorbed or released radially form can be written in dimensionless variables as:

(21) $$\frac{\partial C_{i}}{\partial T}\;=\;\frac{1}{R^{2}}\frac{\partial }{\partial T}\left(R^{2}\frac{\partial C_{i}}{\partial R}\right)\;=\;\frac{\partial^{2}C_{i}}{\partial R^{2}}\;+\;\frac{2}{R}\frac{\partial C_{i}}{\partial R}$$

where *R* represents the relative position within the sphere, and its value is defined as the actual distance from the center (r) over the sphere radius *a*. With respect to time, again the dimensionless variable *T* is given by a relationship among time, the diffusion coefficient of the sorbate in the polymer and the sphere radius:

(22) $$R\;=\;\frac{r}{a};T\;=\;\frac{D_{i}t}{a^{2}}$$

**Figure 6:**
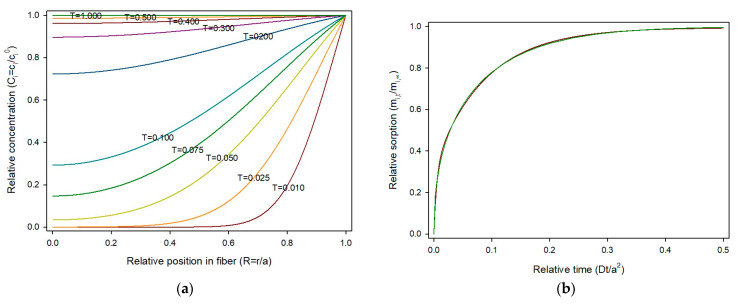
(**a**) Concentration profile of sorbate *i* across the sphere radius over time in dimensionless variables; (**b**) relative sorption in a sphere over time in dimensionless variables. Red line: Equation (22); green line: curve fitting.

Equation (21) was used to obtain the theoretical evolution of sorbate concentration in a sphere. For calculations, the sphere radius was divided in 50 parts (*δR* = 0.02). As noted for fibers and films, smaller finite element for time (*δT*) improve accuracy but increase calculation time. To avoid fluctuations the maximum value for *δT* should be *δT* < 0.5·*δR*^2^ [8]. Thus, *δT* = 0.0001 was selected. Concentrations at each *iδR* point from 0 to 1 (from center to external surface) at times ranging from 0 to 1 were obtained and the concentration profiles at various *T* are shown in Figure 6a.

The amount of sorbate present in the sphere can be calculated by summing the quantities in substance present in the spherical crown volume limited by (*i* + *1*)*δR* and *iδR*, according to Equation (23).

(23) $$\frac{q_{t}}{q_{e}}=\sum_{i=0}^{n-1}\frac{c\left(t\right)}{c\left(\infty \right)}\cdot \frac{4\pi }{3}(R_{i+1}^{3}-R_{i}^{3})$$

The universal sorption profile in a spherical material is presented in Figure 6b. Sorption values were computed for 10,000 time steps (*δT* = 0.0001) and fitted with using the fitting tool of Sigmaplot v16 using the “Exponential rise to maximum, double, 4 parameter” model, as carried out for previous geometries. The fit was excellent, with a R^2^ = 0.9998, being the data indistinguishable as can be observed in Figure 6b. The fitting equation was:

(24) $$\frac{m_{i,\;t}}{m_{i,\infty }}\;=\;0.3251\left(1-e^{-193.8\left(\frac{D_{i}t}{l^{2}}\right)}\right)\;+\;0.6693\left(1-e^{-11.217\left(\frac{D_{i}t}{l^{2}}\right)}\right)$$

This expression presents explicitly the relative gain of solute *i* in a sphere as a function of time with only one unknown, *D_i_*. Thus, converting the gain values ($m_{i,\;t}$) in relative values by estimating the equilibrium gain ($m_{i,\infty }$), and converting the time in dimensionless time by using the sphere radius and a guess value for *D_i_*, by numerical iteration, the value of *D_i_* can be calculated. Excel Solver tool (Microsoft Office 16) was used to obtain an estimation of *D_i_*, by minimizing the squared differences between experimental data and Equation (24).

**Figure 7:**
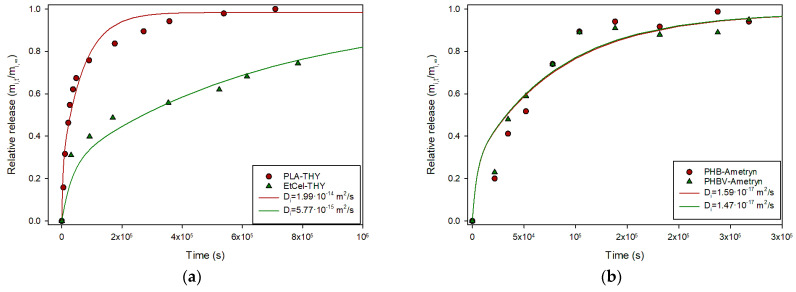
(**a**) Relative release of thymol from PLA [20] and ethyl cellulose spheres [21]; (**b**) Relative release of Ametryn from PHB and PHBV spheres [22].

As in previous sections, data from the literature were collected and tested with this explicit solution. Spherical PLA particles (193 µm diameter) containing thymol (produced by emulsion) were exposed to PBS (phosphate-buffered saline), a water-based salt solution commonly used to maintain pH and osmolarity in biological systems, and the release kinetics were measured and characterized by fitting to a semiempirical power law model [20]. Data from this reference was submitted to the described protocol and the result is presented in Figure 7a. As it can be seen, Equation (24) provided a good description of the experimental data with a *D_i_* value of 2.0·10^−15^ m^2^/s. Similar results were obtained for thymol release from spherical particles of ethyl cellulose (see Figure 7a) [21]. Grillo et al. prepared biopolymeric microspheres containing Ametryn by emulsion in order to ensure a controlled herbicide release, and studied its release, analyzing kinetics with the semiempirical Peppas model [22]. The experimental data were collected and tested with Equation (24). Although the authors considered the process non-Fickian, Equation (24) provides a good description, as shown in Figure 7b.

### 3.4. Recommendations for Using These Equations

One of the most critical aspects when applying the expressions presented in this manuscript (Equations (14), (20) and (24)) is the estimation of *m_i_*_,∞_, since these equations model the evolution of relative sorption. Therefore, experiments should include data from sufficiently long exposure periods to confirm that the mass transfer process has reached completion. When the process is so slow that experimental determination becomes impractical, an approximate value can be obtained through mathematical extrapolation. In fact, the Solver tool of Excel can provide a simultaneous estimation of *m_i_*_,∞_ value and *D_i_*.

Another important consideration is the average thickness or radius of the sample. Typically, thickness values are obtained as the arithmetic mean of multiple measurements due to variability. However, in the estimation of $D_{i}$, the equation includes thickness in the denominator, meaning that as thickness decreases, transfer becomes faster. In this mathematical context, using the arithmetic mean is not recommended. Instead, an effective thickness ($l_{\mathrm{eff}}$) can be estimated by measuring volume and area, although this is not always feasible. A simpler and recommended alternative is to calculate the harmonic mean, defined as:

(25) $$\frac{1}{l_{eff}}=\sum_{i=1}^{n}\frac{1}{l_{i}}$$

where *n* is the number of measurements taken and every $l_{i}$ the individual measurement.

Finally, when using the Solver tool, an initial guess that is too far from the actual value may lead to convergence to an incorrect local minimum. A preliminary plot of the data can be very helpful in avoiding this issue.

## 4. Conclusions

This work presents a comprehensive theoretical framework for describing the sorption and release kinetics of substances in polymeric materials with planar, cylindrical, and spherical geometries. By applying dimensionless variables and finite-difference numerical solutions to Fick’s second law, universal profiles for mass transfer were obtained for each geometry. These profiles were successfully fitted to explicit analytical expressions based on double-exponential models, enabling a straightforward and accurate estimation of diffusion coefficients from experimental data.

The proposed approach overcomes the limitations of classical analytical solutions, which are restricted to planar systems under ideal boundary conditions. The methodology presented here is broadly applicable to a wide range of polymer-based systems, including films, fibers, and microspheres, which are increasingly employed in packaging, biomedical devices, controlled-release formulations, and environmental technologies. Validation with literature data demonstrated excellent agreement between predicted and reported diffusion coefficients, confirming the robustness and versatility of the model.

Overall, this study provides a practical and universal tool for characterizing mass transfer in polymeric materials, facilitating the design and optimization of functional systems in which controlled sorption or release is critical. This could accelerate the implementation of active devices, adapting their design to specific products and applications.

## Data Availability

The original contributions presented in this study are included in the article. Further inquiries can be directed to the corresponding author.
